# The Microbial Resource Research Infrastructure MIRRI: Strength through Coordination

**DOI:** 10.3390/microorganisms3040890

**Published:** 2015-11-18

**Authors:** Erko Stackebrandt, Manuela Schüngel, Dunja Martin, David Smith

**Affiliations:** 1 Microbial Resource Research Infrastructure (MIRRI), c/o Leibniz Institute Deutsche Sammlung von Mikroorganismen und Zellkulturen (DSMZ), Braunschweig 38124, Germany; E-Mails: msc13@dsmz.de (M.S.); dma@dsmz.de (D.M.); 2 Centre for Biosciences and Agriculture International (CABI), Egham, Surrey TW20 9TY, UK; E-Mail: d.smith@cabi.org

**Keywords:** bioeconomy, biological resource centres, global biological resource centres, MIRRI, microbial resource centres

## Abstract

Microbial resources have been recognized as essential raw materials for the advancement of health and later for biotechnology, agriculture, food technology and for research in the life sciences, as their enormous abundance and diversity offer an unparalleled source of unexplored solutions. Microbial domain biological resource centres (mBRC) provide live cultures and associated data to foster and support the development of basic and applied science in countries worldwide and especially in Europe, where the density of highly advanced mBRCs is high. The not-for-profit and distributed project MIRRI (Microbial Resource Research Infrastructure) aims to coordinate access to hitherto individually managed resources by developing a pan-European platform which takes the interoperability and accessibility of resources and data to a higher level. Providing a wealth of additional information and linking to datasets such as literature, environmental data, sequences and chemistry will enable researchers to select organisms suitable for their research and enable innovative solutions to be developed. The current independent policies and managed processes will be adapted by partner mBRCs to harmonize holdings, services, training, and accession policy and to share expertise. The infrastructure will improve access to enhanced quality microorganisms in an appropriate legal framework and to resource-associated data in a more interoperable way.

## 1. The Need to Safeguard Microbial Resources

Based on molecular data, estimates on the number of prokaryotic cells on the planet Earth is somewhere around 5 × 10^30^ [1]. Naturally, estimates on the number of species are much lower, ranging between 6 × 10^5^ [2] and 10^7^ [3] for fungal genomo-species, and up to 10^7^ [4] prokaryotic genomo-species. In reality, the number of named species is significantly lower, around 13,500 for prokaryotes [5] and around 365,300 for yeast and fungi as listed in Mycobank [6].

Scientists, from the very early dawn of microbiology, deposited microbial strains in academic culture collections (CC) for further availability, first to peers from the medical field (from 1875 on), later to those working in agriculture, food and in ecology and physiology (from 1915 on), yet later working in genetics (from 1940 on) and much later for exploitation by the bioindustry (from 1950 on) [7,8]. When the first academic collections became too large to be handled, public collections with their own dedicated staff, often with governmental or academic support, were created worldwide, such as those in the USA (ATCC, ARS), Japan (JCM), and several throughout Europe (e.g., CBS, NCTC, CIP (today CRBIP), CABI [formerly IMI, CMI]). With the evolving bioindustry, the establishment of microbial culture collections witnessed a renaissance (to name a few: NCIMB, BCCM/LMG, DSMZ, CCUG, CECT); this was followed by the recognition of the need to conserve at least a fraction of the enormous wealth of microorganisms from nature for future potential application (e.g., collections in South America, Australia and above all in East Asia were established). At present, 709 collections are registered in the World Data Centre for Microorganisms (WDCM) [9] housing about 1,070,000 bacteria and about 747,000 fungi and yeasts. The number of resources deposited in the listed 220 European collections registered with the WDCM is about 795,000. The abbreviation mBRC (microbial domain biological resource centre) will be used in the following for collections that are run under quality-driven management processes according to OECD guidelines [10].

Besides the availability of well-maintained microbial resources in public CCs and mBRCs, the number of resources isolated world-wide in the context of research projects, deposited in laboratory collections of unknown quantity and quality, most likely outnumbers the number of resources available from culture collections to an unexplored extent. Of these, a minute fraction will be described as new species and will subsequently be available from public collections. Another portion will be included in scientific papers and should, in theory, at least be available to peers upon request if the journal recommendation for sharing resources is followed (the practice, however, indicates, that it is not followed). This leaves a vast number of strains in research collections which are worth maintaining as they represent unique specimens of scientific and technological value. ‘Key strain’ criteria have been identified which should facilitate the recognition of those strains to be deposited in public collections [11].

## 2. The Bottleneck

Public collections play an important role in maintaining and providing microbial resources to scientists from academia and industry; they acquire resources and respond to users need individually. Traditional in-house research strength, national mandate and national cooperation usually determine the breadth and scope of microorganisms collected. As a result, even within a narrow geographic region such as West Europe, dozens of public collections are concentrated which offer to their users a significant overlap in their portfolio, including range of microorganisms, expert knowledge and technical skills. Public funds are used to duplicate effort with little attempt to work more cost effectively by bundling common interest to better serve the user clientele.

Attempts to increase the number of microbial strains in public mBRCs and CCs with strains selected from academic collections immediately points to the emergence of a tightening bottleneck, as, unfortunately, many of the larger and internationally recognized centres do not collaborate with smaller collections; they struggle to keep pace with the mandatory deposition of the increasing number of prokaryotic type strains; and most are not prepared (e.g., infrastructure, personnel, mandate) or even willing to accept a higher workload accompanying the increase of non-type strain and reference holdings. These additional accessions would add an enormous financial and logistical burden to each collection that can only be alleviated by a combination of shared accession policy and strategy, and an expansion of finances, personnel and infrastructure.

In times of declining financial resources, public collections should take the opportunity to establish a new organizational structure to meet the challenges of the microbiological revolution. Rather than focusing on the accession of resources through the glasses of traditional and momentary strength, resource centres should complement each other through the development of strategies for harmonized international acquisition and networking, for improved collection-provider and collection-user dialogs, and for streamlined compliance to international rules and regulations.

## 3. The European Microbial Resource Research Infrastructure (MIRRI)

European mBRCs have a long tradition of giving access to their products, services and expertise to national, regional and international academic and public service user clientele. Among others, they worked together in EU-funded projects offering their strain catalogues on a common platform (www.cabri.eu), adhering to common quality standards and best practices (www.ebrcn.eu) and assessing working and financial models of mBRCs (www.embarc.eu) [12]. Access has also been granted to R & D projects in isolation, as well as to topics related to identification and preservation which facilitated the mutual understanding of individual interests, mode of actions and national policy regimes.

The consequence of these collaborations, based upon previous evidence of successful cooperation and on the results of the Global Biological Resource Centre Network (GBRCN) pilot project [13] was that major European mBRCs submitted the MIRRI project to the European Commission (EC). MIRRI was placed on the European Strategy Forum for Research Infrastructures (ESFRI) roadmap in 2010 and the EC funded preparatory phase project has been running since 2012. MIRRI complements the European landscape of ESFRI infrastructures in the medical (e.g., ECRIN, INFRAFRONTIER, and BBMRI) and biological fields (EMBRC, EU-OPENSCREEN, ERINHA, ISBE) and works with them in the BioMedBridges project and H2020 projects (e.g., EMBRIC, Ritrain, CORBEL). Now, in its final year of the Preparatory Phase, MIRRI has achieved most of its envisaged objectives and is likely to be funded long-term by a combination of state contributions, membership fees and a range of revenue lines. Today, MIRRI has 16 partners and 27 collaborating parties from 19 countries.

It is the vision of MIRRI to be a unique pan-European high-performance platform adding value to known and unknown microbial biodiversity and exploiting novel sources and knowledge to discover and disclose this for the bio-economy and bioscience. MIRRI will address and explore delivery of solutions to societal challenges, such as:

- Fundamental uncertainty in knowledge about where the latent value in microbial resources lies;
- The significant gaps in the available resource base as well as gaps in the data and in taxonomic expertise that underpins resources;
- The need for microbial diversity services to be trusted and sustained;
- Places of origin needing to be able to capture value and benefit from their microbial resources;
- The quality and reproducibility of microbial science need significant improvement.

## 4. How MIRRI Will Be Organized: The Governance Structure

As an individual mBRC cannot present global solutions to microbiological needs on its own, a coordinated approach by a consortium of national mBRCs guided by their stakeholders is required. Likewise, no single country offers a complete coverage of microbial diversity and associated services. Therefore an overarching European organization bringing together distributed national networks, the MIRRI structure, is required to make best use of the current capacity, bridge gaps and address the needs of biotechnology today.

In order to make MIRRI independent from individual national interests and influences, it is envisaged to implement MIRRI under the legal entity of an ERIC (European Research Infrastructure Consortium), which is not bound or connected in any way to any one of its members’ mBRCs. European Member States, along with EU Associated Countries, Third Countries or Intergovernmental Organizations, will be invited to sign the MIRRI-ERIC and thus accept the MIRRI-ERIC Statutes. These statutes set out the main provisions for the governance and management of the MIRRI-ERIC and for its services, activities and operations. The main obligations of Member States are:

- to send a representative to the MIRRI Assembly of Members;
- to provide direction in delivery of appropriate outputs; and
- to provide funding aimed at developing capacity and quality at the national level.

**Figure 1:**
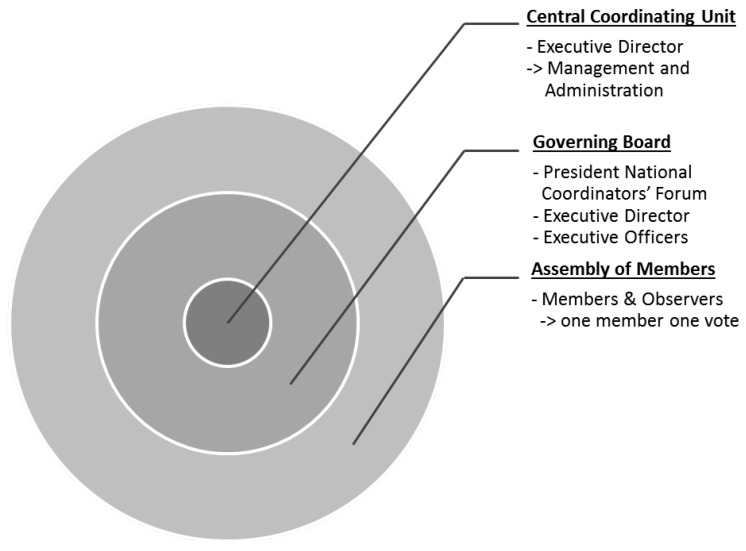
Elements of MIRRIs’ administration.

The three main elements of the MIRRI structure are (Figure 1):

- the Central Coordinating Unit (CCU);
- the Governing Board; and
- the Assembly of Members.

The governance structure envisages an organisational structure with four levels in which the different tasks of (1) Assembly of Members (Member State representatives), the decision making body; (2) the Executive Director of the MIRRI-ERIC and CCU staff; (3) the MIRRI National Node coordinators; and (4) the directors of national mBRCs are realized. Advisory Boards and representatives of the user community constitute additional bodies.

## 5. The Central Coordinating Unit (CCU)

**Figure 2:**
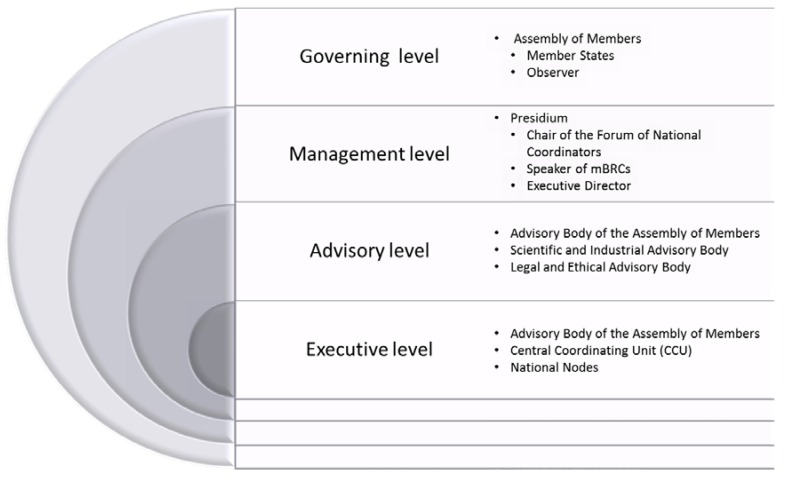
MIRRI’s Steering Elements.

The MIRRI-ERIC will be steered at four levels (Figure 2) receiving decisions and direction from the Assembly of Members at the governing level. The management level under advice of the Advisory Bodies will translate these into action. MIRRI-ERIC will operate on the basis of a central organization, the CCU, hosted in one of the Member States.

## 6. The National Nodes (NN)

The NN can be at a single national mBRC or be a National Network represented by a legal entity appointed by the Member State. Its affiliation to an established structure, such as a national mBRC or a funding body, is up to Member State. Each NN will have a coordinator who would accompany the Member State representative at the MIRRI Assembly of Members. The National Coordinator’s role is twofold: firstly it will act as the main liaison between the MIRRI-ERIC and the National Node. National Coordinators are responsible for their country to follow the Assembly of Members’ policies and strategies for the development and exploitation of the research infrastructure to solve infrastructure problems and European research community needs and challenges. A National Coordinators’ Forum of all National Coordinators will be established to ensure the implementation of the strategies laid out by the Assembly of Members. Secondly, NNs will facilitate user services via the collaboration of the respective national stakeholder communities such as universities, bio-industries, policy makers and scientists to access microbial knowledge.

## 7. mBRCS and National Networks

Within each Member State the mBRCs (either individuals or a network of members) in the NN will sign up to the MIRRI Partner Charter, which will include criteria and specific commitments to data provision and policy adherence. These mBRCs implement the MIRRI work programme as adopted by the Assembly of Members and provide a common gateway to available resources and expertise. The individual mBRCs maintain their own legal status. However, the current independent mBRC policies and managed processes will be adapted by partner mBRCs to harmonize holdings, services, training, and accession policy and to share expertise. Furthermore, by signing the MIRRI Partner Charter, member mBRCs agree to have a part of their activities controlled by the CCU through National Nodes, such as provision of user access and expertise, annual increase of holdings, and linking data to resource catalogues.

## 8. The MIRRI Offer

MIRRI itself is not attempting to build industrial capacity internally but rather to approach bioindustry to build partnerships to expand the current range of microbial products. This will be initiated through an improved data offer, releasing the potential of organisms and creating the necessary partnerships. MIRRI will significantly increase opportunities by coordinating efforts providing a broader range of resources, data and expert services and this is evidenced by the extensive list of supporting companies. Most mBRCs are supported by state governments or academic institutions and any significant revenue generating activities may jeopardize this core funding support. Communication within the CCU will seek to intensify partnerships with SMEs and multinational companies in the long run; private-public partnerships are a logical prerequisite for some MIRRI partners. MIRRI partners can offer joint development projects, targeted isolation and screening for secondary metabolites, technology validation programs to vendors and suppliers, assay developers and instrument builders. MIRRI mBRCs and industrial laboratories face common problems which could be solved by synergistic collaborations such as mutual access to user communities, exchange of best practices, exchange of tools and logistics, shared training and education and appointment of students to their staff. A larger portion of the budget will come from such partnerships as well as materials, special construction and instruments and in kind contributions and exchange of personnel. MIRRI will take these considerations into account through a technology transfer (TT) officer in the CCU to lobby and engage with TT-agencies, educating and hiring people with experience in industrial processes, patenting, licensing, and contracting within individual mBRCs and to coach scientists.

A formal governance structure is necessary for any ESFRI infrastructure to receive legal status, which in turn will guarantee a centralized harmonization of hitherto divergent policies and interests of members. Equally important, however, is the agreement of member mBRCs on common goals to improve the offer to its user clientele from a wide spectrum of science areas. MIRRI will remove fragmentation in resource and service availability and focus on fundamental needs and challenges; this means that MIRRI

- aims to pool existing information on microbial systems, sort them and make them more accessible to users. The idea is to create a user- and quality-driven virtual platform (see below), resulting in a single point of entry for microbial raw material and their associate data, expertise, legal advice and training;
- focuses on the expertise and capacities of participating mBRCs to provide the specific resources to facilitate discovery of solution as outlined in the Sustainable Developmental Agenda and the Sustainable development Goals [14], fostering sustainable economic growth and transformation and promoting sustainable consumption and production. To fight the loss of expertise well-structured training in different fields of modern microbiology will be offered;
- provides the services, including training to access new microbial diversity to support the bio-economy, based on a quality management system which increases the reliability of available organisms and associated data;
- supports the mandatory deposition of key resources included in the scientific literature to safeguard valuable biological material for improving the credibility of science. Costs for resource acquisition and its maintenance are high and generally not covered for all strains worth maintaining for long-term. Many resources, such as cyanobacteria, anaerobes, chemolithotrophs and recalcitrant bacteria present a challenge to collections and additional financial support might be needed to maintain such resources.

In practice MIRRI will be a strong partner in responding to user needs and demands by

**Figure 3:**
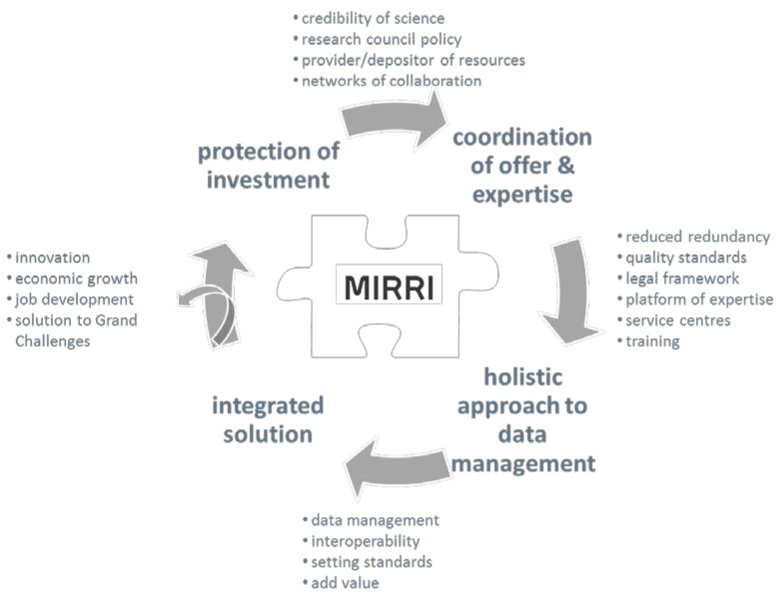
Elements of the MIRRI offer to users.

- facilitating legally protected and regulative compliant access to resources and associated data in mBRCs to maintain a comprehensive supply of biological material in keeping with the demands of the research community by
  - creating a legal operational framework for access to microbial resources which is in full compliance with the Nagoya Protocol [15]. This provides a safe legal background for using microbial resources for the bio-economy; a common acquisition policy will be devised to increase capacity and to engender trust in order to facilitate access to materials from countries with mega-diversity;
  - linking member mBRC holdings with contextual data and publicly available data generated on these microorganisms;
  - ensure that key reference strains from publications are available for the furtherance of science; MIRRI supports the mandatory deposition of key resources included in the scientific literature to safeguard valuable biological material for improving the credibility of science;
- providing expert and technical platforms (Figure 3) to enable researchers to carry out in-house research on mBRC holdings and to offer well-structured training in different fields of modern microbiology to fight the loss of expertise by
  - pooling existing information on microbial systems, sorting them and making them more accessible to users. The idea is to create a user- and quality-driven virtual platform, resulting in a single point of entry for microbial raw material and their associate data, expertise and legal advice;
  - focusing on the expertise and capacities of participating mBRCs to provide the specific resources to facilitate discovery of solutions to grand challenges and Sustainable Development Goals like, among others,
  - achieve food security and improved nutrition, and promote sustainable agriculture;
  - ensure healthy lives and promote wellbeing for all at all ages;
  - take urgent action to combat climate change and its impacts;
  - protect, restore and promote sustainable use of terrestrial ecosystems, sustainably manage forests, combat desertification and halt and reverse land degradation, and halt biodiversity loss;
  - use oceans, seas and marine resources for sustainable development.

MIRRI will offer assistance horizontally to members of the MIRRI consortium to improve their performance as well as vertically to stakeholders at the national and international level. As most MIRRI mBRC partners run under a quality management system or Best Practice guidelines according to the OECD definition, the continuous improvement process aspect, including training of managers and curators, is an intrinsic element of mBRC operations. At the level of the partner mBRCs, this includes the option to train curators in those techniques and methodologies which are deemed important for harmonized quality management and development of staff within individual mBRCs. Attempts to increase the linkage of resources with associated data and the interoperability of data across members must be established on firm data management. MIRRI will not only assist their members and other collections belonging to the European Culture Collections’ Organisation to address their knowledge, operational and management issues, but will reach beyond Europe to liaise with other regional infrastructures with respect to compliance to legal matters (e.g., Nagoya Protocol).

## 9. MIRRI’s Collaborative Working Environment Platform

In order to facilitate outreach and knowledge transfer, the CCU envisages establishing a Collaborative Working Environment (CWE) platform. MIRRI considers this technical solution as a promising and powerful instrument for the promotion of research and innovation, for the increase of competitiveness and subsequently for the creation of economic growth and job creation. In the favourable and dynamic environment of MIRRI, innovative researchers and companies can develop, *i.e*., accelerating development of their business, by interacting with different innovation actors across sectorial boundaries. The central elements of the MIRRI CWE platform are expert clusters which provide a powerful instrument to reap the greatest benefit from the research infrastructure:

- expert clusters address topics aligned with the stakeholder demands;
- they bring together the most appropriate pool of experts for an extensive range of responses and knowledge;
- the cluster tools are selected for an optimally and efficient intercommunication and exchange of knowledge and experience;
- they provide highly automated request matching for a multilayer reply to incoming requests.

The vision is to design gates leading to different clusters which will be programmed to facilitate cross-talk between groups/clusters, thus facilitating the knowledge transfer to users. The offer can be compared to a customer entering a department store. Originally focused on a pre-set errand, the offers and displays from other windows/entry gates make it tempting to browse for additional options and opportunities. For the user of the CWE this means that rather than searching a large number of web-sides individually, the CWE, based on a specific search term, would generate a highly automated request matching delivering a multilayer reply to incoming requests. Examples of gates are:

1. Gate to Collaboration & Experts to respond to and solve infrastructure problems and European research community needs and challenges, e.g.,
  - legal issues
  - taxonomy and relevant technologies
  - application-driven, providing an opportunity for experts to be brought together to provide microbial solutions;
  - quality management;
  - new technology application and coordination of use;
2. Gate to Resources & Data e.g.,
  - information on all biological resources within Europe or even worldwide;
  - accessibility to high-quality information including strain metadata and literature information, linked to individual resources upon request;
  - preservation technology to broaden the range of organisms stored improving reliability and reproducibility.
3. Gate to Training & Education, e.g.,
  - on-site training;
  - e-learning and academic curricula.

As of yet, the CWE is an IT-based implementation concept for a community/consortium project. This means in general that it will be an evolving topic, as MIRRI itself will be evolving. Not all elements/parts of the CWE are presently defined, but those identified show the appropriate way to establish MIRRI services and benefits on a single platform and enable MIRRI to run with limited financial and human resources. The CWE model will use state-of-the-art IT-tools, which other communities are already using successfully on a single platform for both internal and external tasks. The use of the CWE platform does not mean that MIRRI acts purely virtual; it simply means that the main operational and most efficient and effective tool of MIRRI to present its services and benefits to the community/stakeholders and to cope with many internal administrative affairs is operated though the platform.

## 10. Envisaged Benefit

Connection of strain data to other relevant data sets to facilitate the generation of knowledge is one of the main missions of MIRRI, following the principles laid down in the report *Riding the wave* explaining *How Europe can gain from the rising tide of scientific data* [16] where the benefits of accelerating the development of a fully functional e-infrastructure for scientific data is described. Delivery of high-quality microbial resources with facilities to data mine associated data sets will facilitate research and accelerate the discovery of new products and stimulate innovative microbial solutions to the Grand Challenges. MIRRI will facilitate this by ensuring interoperability between microbial strain data and other relevant data sets such as DNA and protein sequences, ecological data, metabolites, geographical data and phylogenetic relationships. MIRRI will deliver a coordinated accession policy to broaden coverage of resources, increase capacity to store and deliver more characterised microorganisms to researchers. MIRRI will seek data holding partners and, through ELIXIR and BioMedBridges initiatives, will ensure interoperability of its community data to facilitate accelerated discovery and innovation, and interaction with other regional networks.

The MIRRI-ERIC will include a proportion of user access to facilities, services and resources; a commitment to take deposits identified in the MIRRI common accession policy; and participation in the expert clusters. mBRC participation in the MIRRI National Nodes will be governed by commitments made in the Partner Charter which will include delivery of high-quality data to agreed standards, participation in capacity-building programmes and a commitment to deliver the MIRRI communication and outreach strategy to stakeholders, particularly to bioindustry.

This will expand the knowledge underpinning research and education, improve efficiency and cost effectiveness in the management of microbial resources and protect investment in publicly funded research. Long-term world-wide access to the biological materials on which the science is based will enable confirmation of results and further work facilitating high-quality research and good science [13]. The research infrastructure will support academic and industrial research with high-quality services and material. The societal impact will be economic improvement and increased employment through the biotechnological discoveries in healthcare, food security and livelihoods that MIRRI will support.

## Abbreviations

ARS	Agricultural Research Service
ATCC	American Type Culture Collection
BBMRI	Biobanking and Biomolecular Resources Research Infrastructure
BCCM/LMG	Belgian Co-Operated Collection of Microorganisms/Laboratory of Microbiology Ghent University
CABI	Centre for Biosciences and Agriculture International
CBS	Centraalbureau voor Schimmelcultures
CC	Culture Collection
CCU	Central Coordinating Unit
CCUG	Culture Collection of the University of Gothenburg
CECT	Spanish Type Culture Collection
CIP	Collection of the Institute Pasteur
CMI	Commonwealth Mycological Institute
CORBEL	Coordinated Research Infrastructures Building Enduring Life-Science Services
CRBIP	Biological Resource Center of Institut Pasteur
CWE	Collaborative Working Environment
DSMZ	Leibniz Institute DSMZ—German Collection of Microorganisms and Cell Cultures
EC	European Commission
ECRIN	European Clinical Research Infrastructure Network
ELIXIR	European Life Science Infrastructure for Biological Information
EMBRC	European Marine Biological Resource Centre
EMbaRC	European Consortium of Microbial Resource Centres
EMBRIC	European Marine Biological Research Infrastructure Cluster
ERIC	European Research Infrastructure Consortium
ERINHA	European Research Infrastructure on Highly Pathogenic Agents
ESFRI	European Strategy Forum for Research Infrastructures
EU	European Union
EU-OPENSCREEN	European Infrastructure of Open Screening Platforms for Chemical Biology
IMI	Imperial Mycological Institute
INFRAFRONTIER	European Research Infrastructure for phenotyping and archiving of model mammalian genomes
ISBE	Infrastructure for Systems Biology in Europe
IT	Information Technology
JCM	Japanese Culture Collection
mBRC	microbial Domain Biological Resource Centre
MIRRI	Microbial Resource Research Infrastructure
NCIMB	National Collection of Marine Bacteria
NCTC	National Collection of Type Cultures
NN	National Node
OECD	Organisation for Economic Co-operation and Development
R&D	Research and Development
RiTrain	Research Infrastructures Training Programme
SME	Small and Medium Enterprise
TT	Technology Transfer
WDCM	World Data Center of Microorganisms
